# Endoscopic papillectomy; a retrospective international multicenter cohort study with long-term follow-up

**DOI:** 10.1007/s00464-020-08126-x

**Published:** 2020-11-06

**Authors:** Jeska A. Fritzsche, Amir Klein, Maarten J. Beekman, Jeanin E. van Hooft, Mayenaaz Sidhu, Scott Schoeman, Paul Fockens, Michael J. Bourke, Rogier P. Voermans

**Affiliations:** 1 Department of Gastroenterology and Hepatology, Amsterdam Gastroenterology Endocrinology Metabolism, Amsterdam University Medical Centers, University of Amsterdam, Amsterdam, The Netherlands; 2 Department of Gastroenterology and Hepatology, Westmead Hospital, University of Sydney, Sydney, New South Wales, Australia; 3 Westmead Clinical School, University of Sydney, Sydney, New South Wales, Australia

**Keywords:** Endoscopic papillectomy, Papillary adenoma, Recurrence, Long-term follow-up

## Abstract

**Background:**

Endoscopic papillectomy (EP) is considered a relatively safe and minimally invasive treatment for papillary adenomas. In the literature a significant risk for local recurrence is described. The aim of this study was to evaluate long-term recurrence rates and time-to-recurrence. Additionally, risk factors for recurrence, malignancy and adverse events were studied.

**Methods:**

This is a retrospective study in consecutive patients with papillary adenomas who underwent EP in two tertiary referral hospitals between 2001 and 2018. Primary outcome was recurrence in patients with at least 1-year endoscopic follow-up. Secondary outcomes were surgery free survival, adverse events, and mortality within 30 days after the index procedure.

**Results:**

A total of 259 patients were found eligible [median age 66 years, 130 male (50.2%)]. Forty-three patients were known with familial adenomatous polyposis (FAP) (16.6%). At least 1-year endoscopic follow-up was available in 154 patients with a total follow-up of 586 person-years and median of 40 months [interquartile range (IQR) 25–75]. Recurrence occurred in 24 cases (15.6%) of which 8 were known with FAP, leading to a recurrence incidence rate of 4.1 per 100 person-years with a median time-to-recurrence of 29 months (IQR 14.75–59.5). Fifty-three patients underwent at least 5-year follow-up, in 6 (11.3%) of them recurrence was encountered after 5 years of which four were known with FAP. No risk factors for recurrence could be identified. Adverse events occurred in 50/259 patients (19.3%). One patient died within 30 days after the procedure. Papillary stenosis occurred in 19/259 (7.3%) of the patients. There were no cases of malignant degeneration during follow-up.

**Conclusions:**

Recurrence after EP occurs in a significant proportion of patients and occurs even 5 years after EP. This emphasizes the need for long-term follow-up. We advise to consider at least 5-year follow-up in case of a sporadic adenoma, unless comorbidity makes follow-up clinically irrelevant.

**Electronic supplementary material:**

The online version of this article (10.1007/s00464-020-08126-x) contains supplementary material, which is available to authorized users.

Adenomas of the major duodenal papilla, also known as papillary adenomas, are rare. Nomenclature in literature sometimes calls these lesions ampullary adenomas. The estimated prevalence is 0.04–0.12% as suggested by autopsy studies [1]. Making the diagnosis of a papillary adenoma requires an expert pathologist since these lesions are frequently overdiagnosed by general pathologists. Papillary adenomas can occur sporadically or in the context of a genetic predisposition, as seen in familial adenomatous polyposis (FAP) [2].

Historically, patients used to present with clinical signs of biliary obstruction and/or pancreatitis [3]. Nowadays, the majority of papillary adenomas are detected at an asymptomatic stage, most likely due to the increasing use and optical resolution of gastroduodenoscopy as well as abdominal imaging as diagnostic modalities [4]. It is believed that papillary adenomas follow the same adenoma to carcinoma pathway as described for colonic adenomas. Up to 30% are expected to progress to invasive adenocarcinoma [5, 6]. Consequently, it is recommended to resect sporadic papillary adenomas once diagnosed regardless of the presence of symptoms.

Endoscopic papillectomy (EP) is considered a relatively safe and minimally invasive treatment method for lesions without significant intraductal extension or malignant degeneration. Therefore, endoscopic resection is preferred over surgical resection methods such as pylorus-preserving pancreatic duodenectomy (PPPD) or surgical ampullectomy, given the high morbidity (up to 52%) and mortality (up to 2.1%) associated with these procedures [7–15]. Although the mortality (0–1%) of EP is very low [14], complications occur in a significant proportion of the patients (19–35%) [11, 16–21]. The most common adverse events are: post-procedural bleeding (5–20%), acute pancreatitis (3–20%), perforation (0–8%), cholangitis (0–7%), and papillary stenosis (0–7%) [11, 16–21].

Furthermore, since EP only removes the intraduodenal proportion of the ampulla of Vater, it does not eliminate the risk of local recurrence as is the case with radical surgery [7]. Recurrence rates up to 21% have been reported with mean follow-up periods varying between 19 and 43 months [11, 17, 18, 20]. However due to the low incidence, large series addressing long-term outcome and recurrence are scarce. Accordingly, there is a lack of consensus regarding optimal duration of follow-up. In general, literature states that surveillance should be performed for at least 2 years after the index procedure [11, 12, 17, 22]. Nonetheless, late recurrences after 2 years of follow-up have been described [11].

The primary aim of this study was to evaluate long-term recurrence rates and median time-to-recurrence after EP. Additionally, complication rates were evaluated and risk factors for recurrence, malignancy and adverse events were studied.

## Materials and methods

### Study design

This is a two-center international retrospective study of consecutive patients with papillary adenomas who underwent EP between 2001 and 2018 at Amsterdam UMC, location AMC, in the Netherlands and between 2007 and 2018 in Westmead Hospital in Sydney, Australia. The study protocol was approved by both institutional review boards.

All patients who underwent EP were identified using endoscopic report databases (Olympus Endobase^®^ in Amsterdam UMC and the local database in Westmead Hospital). Patients were excluded when pathology showed another diagnosis than adenoma or when resection had been previously attempted elsewhere. Patients who underwent further follow-up at the referring hospital instead of the treating institution were asked active permission to request this information. When endoscopic follow-up of at least 1-year was not available, patients were excluded for primary endpoint analysis and were included for secondary outcomes (adverse events and mortality within 30 days) only.

The complications and short-term follow-up of the Westmead cohort has been previously described by Klein et al. [23]. In this study we will describe further follow-up in these patients.

### Data collection

Patient and lesion characteristics, endoscopic procedural details, pathology results, complication and follow-up data were recorded from either the endoscopy database or retrieved from electronic or paper medical records. Further follow-up data was requested from the referring hospital when available. Data was collected by different researchers (MBe, AK, MS, SS) and cross-checked and combined in a database by another researcher (JF).

### Procedure

All patients were re-evaluated prior to resection at the treating institution. Additional imaging was performed at the discretion of the treating physician. All procedures were performed by an experienced interventional endoscopist or senior fellow under direct supervision. Lesion size was estimated using an open snare of known size, a lateral spreading lesion (LSL) was defined as an extension of the polyp >10 mm on the free duodenal wall. En bloc resection was performed when possible. Resection was performed with fractionated current at the plane of the duodenal wall during the whole study period using a smart electrosurgical generator. Snare type and the use of submucosal injection was left at the discretion of the endoscopist. In patients without a known pancreas divisum a stent in the pancreatic duct (PD) was routinely placed to reduce the risk of post-procedural pancreatitis. Since 2013 rectal NSAID was administered to reduce the risk of pancreatitis as well. The placement of a stent in the common bile duct (CBD), either plastic or a fully covered self-expandable metal stent (FCSEMS), after resection was left up to the decision of the treating endoscopist. In general, FCSEMS was placed if a CBD-stent was indicated because of ongoing bleeding from the papillary region, concerns for (micro) perforation after resection or in case of residual tissue to facilitate the direct inspection of the distal CBD in the next procedure. For other indications a plastic stent was generally preferred. Patients were admitted for at least 1 night of observation or the procedure was performed in the early morning and patients were sent home after 2–4 hours of uncomplicated observation.

### Surveillance protocol

In case a PD-stent was placed patients underwent a plain abdominal film 7–14 days after the procedure. If the stent had not migrated spontaneously, it was removed endoscopically. First follow-up to check for residual tissue was regularly performed within 4–6 months after the initial procedure. Subsequently, follow-up was performed every 6–12 months. Duration of follow-up and, if applicable, timing of removal of the CBD-stent was at the discretion of the threating physician.

### Outcome parameters

The primary outcome was recurrence in patients with at least 1-year endoscopic follow-up available. Recurrence was defined as histological proven recurrence after at least one recurrence free follow-up procedure or encountered after 1 year without known surveillance when initial resection was considered complete. In case no biopsies were taken, endoscopic treatment of a lesion suspected for recurrent adenomatous tissue was considered recurrence as well.

Secondary outcomes were recurrence rates in patients with at least 5-year follow-up, surgery free survival rates, adverse events, and all-cause mortality within 30 days after index procedure. Surgery free survival was calculated by assessing the need for surgery, consisting of all patients that underwent surgical resection of the ampulla as well as all patients with a proven malignancy after initial resection who did not undergo surgery due to age or comorbidity. Adverse events included pancreatitis, post-procedural bleeding, perforation, and cholangitis within 30 days of the index procedure, and papillary stenosis during follow-up. Pancreatitis was defined, according to the revised Atlanta criteria, as the presence of two of the three following symptoms (a) new onset or worsening of upper abdominal pain, (b) elevation of pancreatic enzymes (amylase and/or lipase) and (c) imaging suggestive for pancreatitis [24]. Perforation of the duodenum or distal bile duct was defined as endoscopic visualization of a perforation or leakage of contrast or free fluid and/or abscesses on commuted tomography (CT) performed after intervention. Post-procedural bleeding after intervention was defined as the need for blood transfusion or endoscopic, surgical or angiographic intervention. Cholangitis was defined, according to the Tokyo Guidelines 2018, as systemic inflammation (fever, shaking chills or laboratory evidence of inflammatory response) and either clinical or laboratory evidence of cholestasis or evidence of cholestasis on biliary imaging [25]. Papillary stenosis was defined as cholestasis with a proven papillary stenosis at cholangiogram during follow-up or dilatation of the pancreatic duct.

### Statistical analysis

Descriptive analyses were performed, and categorical data are presented as frequency with percentages and continuous variables as mean with standard deviation (SD), in case of normal distribution, or median with interquartile range (IQR) in case of a non-normal distribution. Logistic regression analysis was performed to identify factors associated with recurrence, complications, and malignancy. Results are presented as odds ratio (OR) with 95% confidence intervals (95% CI). Statistical significance was defined as a *p* value < 0.05. Surgery and recurrence free survival was estimated by drawing a Kaplan–Meier survival curve. All statistical analysis was performed using IBM SPPS Statistics 25.

## Results

### Baseline characteristics

**Fig. 1:**
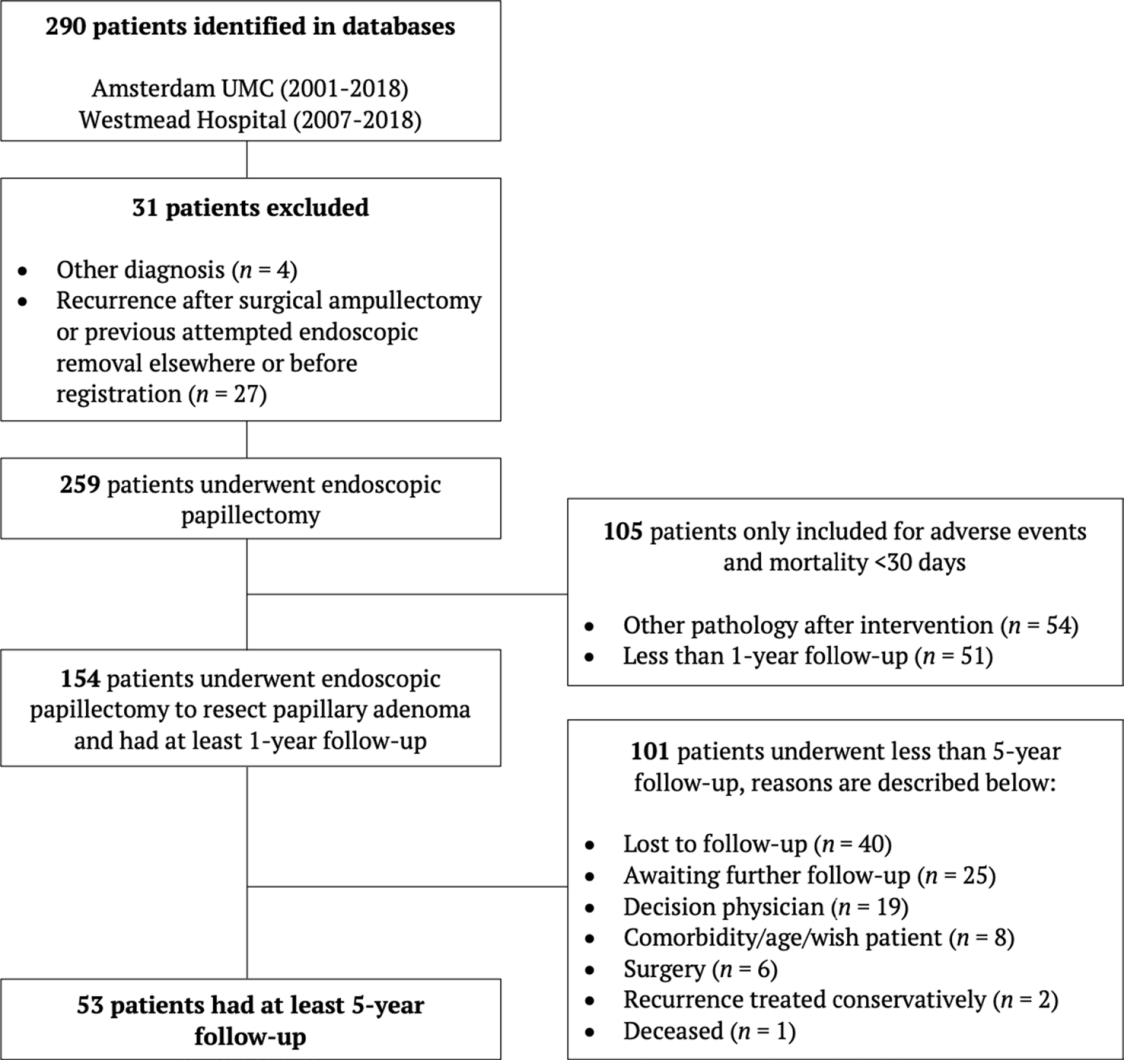
Screening and enrolment flowchart

A total of 259 patients were found eligible for analysis. Follow-up data of at least 1-year was available in 154 patients. The remaining 105 patients were included for the secondary endpoints only (Fig. 1).

Baseline patient characteristics are summarized in Table 1. The median age of the patients was 66 years (IQR 55–76) and 130 (50.2%) of them were male. Forty-three patients (16.6%) were known with FAP, and in 41 of these patients (95.4%) papillary adenoma was detected during surveillance endoscopy. In 113 patients (43.6%) the papillary adenoma was an incidental finding discovered during upper gastrointestinal endoscopy or abdominal imaging. Symptomatic patients presented with jaundice (*n* = 14, 5.4%) or pancreatitis (*n* = 10, 3.9%). In 40 cases (15.4%) the patient was examined because of cholestasis without jaundice. The reason for presentation was unknown in 41 patients (15.8%).

**Table 1:** Baseline characteristics

Characteristics	*N *= 259
Age (years)—median (IQR)	66 (55–76)
Male sex—no. (%)	130 (50.2%)
FAP—no. (%)	43 (16.6%)
Presenting symptoms—no. (%)	
Coincidental finding	113 (43.6%)
Unknown	41 (15.8%)
FAP surveillance	41 (15.8%)
Cholestasis without jaundice	40 (15.4%)
Jaundice	14 (5.4%)
Pancreatitis	10 (3.9%)
Lesion size (mm)—mean ± SD^a^	21.07 ± 1.79
Lateral spreading—no. (%)	72 (27.8%)
Intraductal extension—no. (%)	38 (14.7%)
Pathology before resection	
LGD	123 (47.5%)
HGD	33 (12.7%)
Adenocarcinoma	5 (1.9%)
Normal duodenal mucosa	4 (1.5%)
Unknown	94 (36.3%)

*FAP* familial adenomatous polyposis, *HGD* high-grade dysplasia, *IQR* interquartile range, *LGD* low-grade dysplasia, *SD* standard deviation

^a^Missing in 115 cases

### Procedural details

Procedural details are summarized in Table 2. Resection was performed en bloc in 153 patients (59.1%). In 206 patients (79.5%) a PD-stent was successfully placed after resection. Reasons to omit a PD-stent was pancreas divisum (*n* = 10, 18.9%) or failed cannulation of the PD (*n* = 26, 49.1%). In three patients (5.7%) the endoscopist decided not to place a stent and in 14 patients (26.5%) the reason was unknown. In 94 patients (36.3%) either a plastic stent (*n* = 67, 71.3%) or FCSEMS (*n* = 24, 25.5%) was placed in the CBD. In three patients the type of stent was unknown.

**Table 2:** Procedural details

Procedural details	*N* = 259
En bloc resection—no. (%)	153 (59.1%)
PD-stent—no. (%)	206 (79.5%)
CBD-stent—no. (%)	94 (36.3%)
Plastic	67 (71.2%)
FCSEMS	24 (25.5%)
Unknown	4 (4.3%)
Pathology after resection—no. (%)	
LGD	164 (63.3%)
HGD	40 (15.4%)
Adenocarcinoma	37 (14.3%)
Other	10 (3.9%)
Normal	4 (1.5%)
Unknown	4 (1.5%)
Hospital stay (days)—median (IQR)^a^	1 (1–2)

*CBD* common bile duct, *FCSEMS* fully covered self-expandable metal stent, *HGD* high-grade dysplasia, *LGD* low-grade dysplasia, *PD* pancreatic duct

^a^Missing in 5 cases

### Lesion characteristics

The mean estimated lesion size was 21 mm (SD 1.79) with a lateral spreading component in 72 lesions (27.8%). In 38 cases (14.7%) limited (less than 1 cm) intraductal extension was suspected during the procedure. Either missed at pre-operative imaging, imaging was not performed or patient was considered unfit for surgical management. Pre-operative pathology was available in 165 patients (63.7%). Showing low-grade dysplasia (LGD) in 125 patients (75.8%), high-grade dysplasia (HGD) in 33 patients (20%), adenocarcinoma in five patients (3%) and normal duodenal mucosa in two patients (1.2%).

In 164 patients (63.3%) pathology after resection showed LGD, in 40 patients (15.4%) HGD. In 10 patients (3.9%) the resected specimen showed pathology other than adenoma or adenocarcinoma e.g., hamartoma, neuro-endocrine tumor or adenomyomatosus. In four patients (1.5%) only normal duodenal mucosa was present in the resected specimen while biopsy prior to resection showed LGD. In four patients (1.5%) the pathology was unknown due to loss of the specimen. Resected adenomas were classified as tubulovillous adenoma (TVA) in 139 (68.1%) and as tubular adenoma (TA) in 57 (27.9%); 8 (3.9%) were unspecified. Adenocarcinoma was present in 37 patients (14.3%) of whom only 19 (51.4%) were considered suspicious based on endoscopic appearance and four (10%) were already diagnosed by pre-resection biopsy. In 18 cases (48.6%) malignancy was present in a TVA, 1 (2.7%) in a TA, and in 18 cases (48.6%) surrounding adenoma was not specified.

### Factors associated with malignancy

Results are summarized in Table 3. The univariate analysis showed that age (OR 1.1, 95% CI 1.046–1.129), cholestasis with (OR 14.0, 95% CI 4.365–44.584) or without jaundice (OR 2.4, 95% CI 1.043–5.389), intraductal extension (OR 2.6, 95% CI 1.123–5.866) and possible incomplete resection (OR 3.4, 95% CI 1.441–7.867) were all associated with malignancy. In multivariable analysis this result could only be confirmed for age (OR 1.1, 95% CI 1.025–1.109) and jaundice (OR 10.1, 95% CI 2.881–35.458).

**Table 3:** Logistic regression analysis for factors associated with malignancy

Variable	Univariate analysis OR (95%CI)	Multivariable analysis OR (95%CI)
Age	**1.09 (1.046–1.129)**	**1.07 (1.025–1.109)**
Lesion size^a^	1.00 (0.967–1.026)	**–**
Jaundice	**13.95 (4.365–44.584)**	**10.11 (2.881–35.458)**
Cholestasis	**2.37 (1.043–5.389)**	2.06 (0.819–5.204)
Intraductal extension	**2.57 (1.123–5.866)**	1.19 (0.431–3.255)
Incomplete resection	**3.37 (1.441–7.867)**	2.56 (0.941–6.993)
TVA	0.52 (0.260–1.055)	**–**

Bold values denote statistical significance at the 5% level

*CI* confidence interval, *OR* odds ratio, *TVA* tubulovillous adenoma

^a^Missing in 115 cases

### Primary endpoint: long-term follow-up

In patients with at least 1-year follow-up, a total follow-up period of 586 person-years and a median of 40 months (IQR 25.75–68) was available. Recurrence occurred in 24 patients (15.6%), leading to a recurrence incidence rate of 4.1 per 100 person-years with median time-to-recurrence of 29 months (IQR 14.75–59.5). Recurrence was histological confirmed in 21 cases (87.5%). Six patients developed a second recurrence 26.5 months (IQR 12.5–40.5) after the first recurrence although resection was considered complete. No significant factors associated with recurrence could be shown (Table 4).

**Table 4:** Logistic regression analysis for factors associated with recurrence in patients with at least 1-year follow-up (n = 154)

Variable	Univariate analysis OR (95%CI)
Sex	0.51 (0.210–1.259)
Age	0.99 (0.959–1.017)
FAP	1.74 (0.678–4.475)
Lesion size^a^	1.02 (0.987–1.051)
Lateral spreading	0.81 (0.311–2.086)
Intraductal extension	0.82 (0.172–3.881)
Piecemeal	0.48 (0.295–1.768)
TVA	0.82 (0.334–2.033)
HGD	0.16 (0.020–1.225)

*CI* confidence interval, *FAP* familial adenomatous polyposis, *HGD* high-grade dysplasia, *OR* odds ratio, *TVA* tubulovillous adenoma

^a^Missing in 115 patients

Recurrence was managed endoscopically in 16 cases (66.7%), in five patients the recurrence was not treated in case of FAP, due to comorbidity or wish of the patient, or treatment was unknown. Three patients were referred for surgical management, two because of non-radical removal of the recurrence and one was referred for a duodenectomy because of FAP (Table 5). No patients developed a papillary carcinoma during follow-up.

**Table 5:** Recurrence in patients with at least 1-year follow-up (*n* = 154)

Primary endpoint	Sporadic (*n* = 117)	FAP (*n* = 37)
Recurrence—no. (%)	16 (13.7%)	8 (21.6%)
Treatment recurrence—no. (%)		
Endoscopic	12 (75%)	4 (50%)
Surgery	2 (12.5%)	1 (12.5%)
Conservative/unknown	2 (12.5%)	3 (37.5%)
Time-to-recurrence (months)—median (IQR)	25.5 (10.25–33.75)	55 (25–72.5)

*FAP* familial adenomatous polyposis, *IQR* interquartile range

**Fig. 2:**
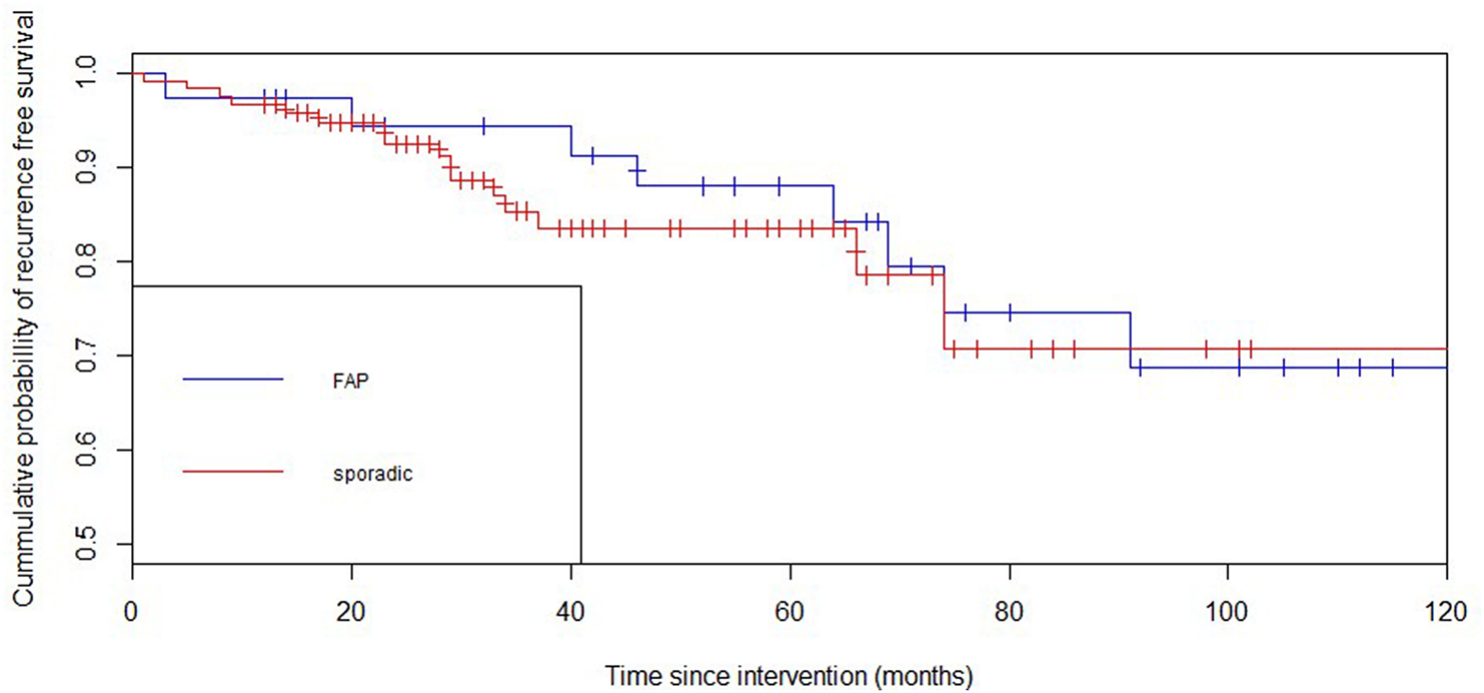
Recurrence free survival in group with at least 1-year follow-up (*n* = 154)

The recurrence free survival rates are shown in a Kaplan–Meier survival curve (Fig. 2). This curve shows an estimated cumulative probability of recurrence free survival after 5 years of 83.4% (95% CI 75–91.8%) in case of a sporadic adenoma and 87.9% (95% CI 76.7–99.1%) in case of FAP (*p* = 0.46).

At least 5-year endoscopic follow-up was available in 53 patients with a total follow-up period of 373 person-years and a median follow-up of 80 months (67.5–104.5). Recurrence occurred in 12 patients (22.6%). The median time-to-recurrence was 55 months (IQR 25.5–72.75). Recurrence occurred after 5-years follow-up in 6 patients of whom at least 5-year follow-up was available (11.3%). Four of these late recurrences occurred in patients known with FAP.

### Surgery free survival

**Fig. 3:**
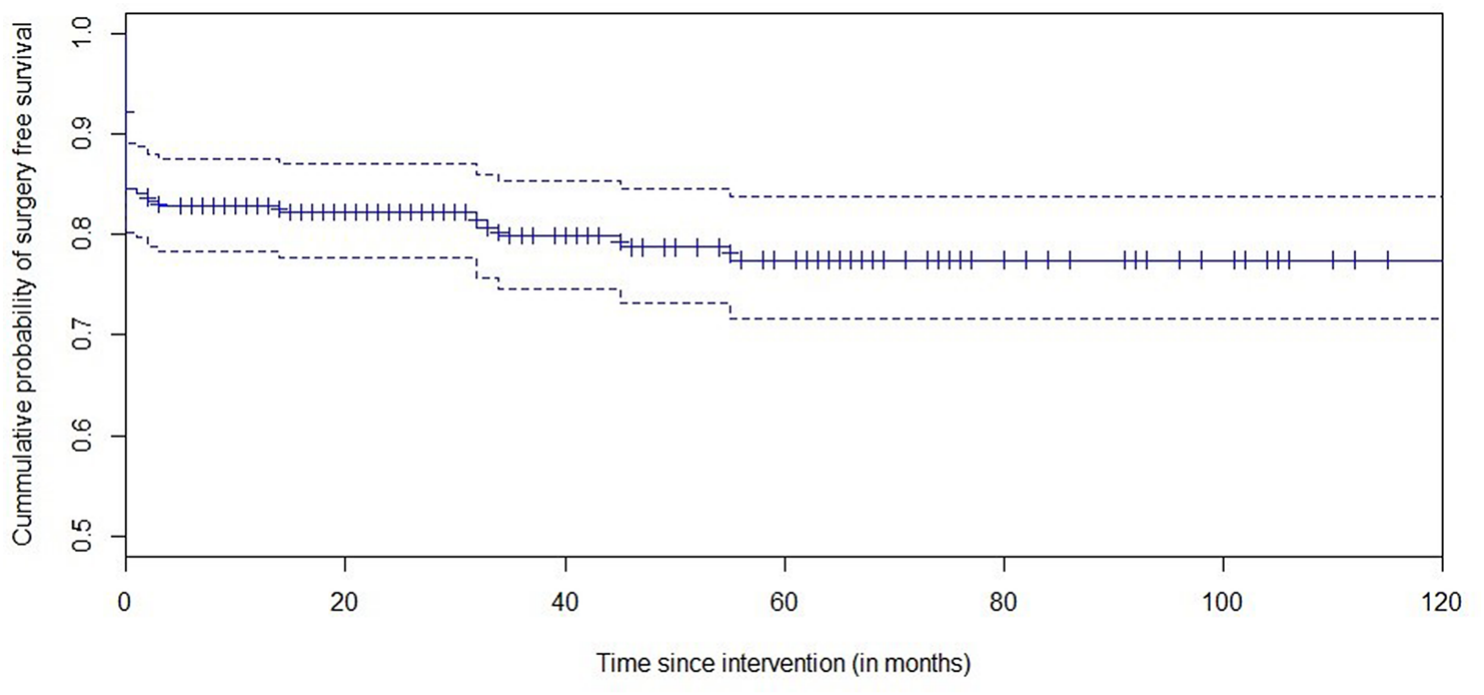
Surgery free survival in all patients (*n* = 259)

Surgery was performed in 31 of 259 patients. Indications included adenocarcinoma in the resected specimen (*n* = 18, 6.9%), non-radical endoscopic removal (*n* = 10, 3.9%) and duodenectomy due to high burden of disease in case of FAP (*n* = 3, 1.2%). Another 19 patients were designated as needing surgery because of adenocarcinoma in the initial resected specimen, however either endoscopic or conservative treatment was preferred due to comorbidity. Figure 3 shows a Kaplan–Meier surgery free survival curve, demonstrating an estimated 77.5% (95% CI 71.4–83.6%) cumulative probability of surgery free survival, which is stable after 5 years.

### Adverse events

The most common short-term complications were post-procedural bleeding (*n* = 29, 11.2%) and pancreatitis (*n* = 18, 6.9%) (Table 6). Data on cholangitis was only available in 146 patients of whom 5 (3.4%) developed cholangitis. No perforations were reported in this study cohort. One patient (0.4%) died within 30 days due to an acute necrotizing pancreatitis after coagulation therapy for post-procedural bleeding. Median hospital stay was 1 day (IQR 1–2). Nineteen patients (7.3%) showed signs of papillary stenosis at cholangiogram during follow-up (Table 6). No risk factors for individual adverse events could be identified (Supplemental Table 1).

**Table 6:** Adverse events

Adverse events	*N* = 259
Complications <30 days—no. (%)	50 (19.3%)
Post-procedural bleeding	29 (11.2%)
Pancreatitis	18 (6.9%)
Cholangitis^a^	5 (3.4%)
Perforation	0 (0%)
Papillary stenosis—no. (%)	19 (7.3%)
Mortality <30 days—no. (%)	1 (0.4%)

^a^Missing in 113 patients

## Discussion

This two-center international study describes long-term follow-up after endoscopic resection of papillary adenomas. Our cohort shows a recurrence rate of 15.6%. The median time-to-recurrence was 25.5 months, and recurrence was found up to 91 months after the index procedure was performed.

The recurrence rate is comparable to published data that showed recurrence rates ranging from 7 up to 21% [11, 17, 20, 26]. However, to our best knowledge, this is the first study including patients with over 3–5 years of follow-up. The longest available prospective follow-up data is 36 months and showed a recurrence rate of 7.2% [17].

Despite the relatively high recurrence rate, invasive surgery such as PPPD was successfully prevented in the vast majority of patients (88%) who were initially treated endoscopically. Furthermore, it is interesting to mention that even though patients developed late recurrences, we observed no case of malignant degeneration of an adenoma which may have consequences for follow-up especially in old and frail patients.

Different studies advise a follow-up period of at least 2 years [11, 12, 17, 22]. However, our study shows that 58.3% of the recurrences occurred after these 2 years, emphasizing the need for longer follow-up. In fact, 25% of recurrences were encountered after 5 years. The majority of these late recurrences occurred in patients known to have FAP. However, no significant correlation between FAP and late recurrence could be shown, probably due to overrepresentation of FAP in the group with long-term follow-up. Expert consensus achieved by recently performed Delphi process concluded that at least 5-years follow-up should be performed after EP since 75% of the international experts agreed on this statement [27]. At this time it is unclear whether surveillance strategy should differ between FAP and sporadic cases. Also, since no differentiation can be made between a second primary adenoma and recurrence, question is if all adenomas encountered after resection should be called recurrence.

Furthermore, this study reports an acceptable morbidity (21%) and mortality (0.4%) rate, comparable with previous studies, confirming the relatively safe character of the procedure [10–12]. Concerning individual adverse event rates, the reported percentage of pancreatitis (6.9%) was relatively low compared to previous literature (3–20%). Although majority of patients were admitted for 1 night, some cases could have been missed because they presented at the referring hospital with a pancreatitis. On the other hand, it could also be the positive effect of standard use of rectal NSAID and placement of a PD-stent after resection. Papillary stenosis, however, occurred in a relatively high percentage of the cases (7.3%) when compared to previous data (0–7%) [11, 16–21].

Previous studies showed that lesion size and the presence of relevant symptoms are factors associated with malignancy [11, 28]. In this study only age and jaundice could be identified as associated factors in multivariable analysis. The data does confirm the low accuracy of biopsy in identifying adenocarcinoma [29]. Additionally, we want to point out that almost 50% of the malignancies were not expected to be malignant prior to resection based on the endoscopic appearance, questioning the accuracy of endoscopic assessment.

This study has some limitations inherited to the retrospective design and the long study period. First, due to the design of the study follow-up was not standardized. E.g. time-to-recurrence could be overestimated because of long (2 years) interval between follow-up procedures. Furthermore, due to the fact that the study was performed in two tertiary referral centers, a proportion of the patients underwent follow-up in the referring center. Where possible this information was retrieved. Nonetheless, 33 patients were lost to follow-up before reaching 1-year surveillance and 73 before reaching 5-years. Consequently, we acknowledge that long-term data could be subject to selection bias considering that patients who are more likely to develop recurrence have probably been followed more extensively. However, as shown by our data as well, it is hard to predict which patients are more likely to develop recurrence. Moreover, since, in contrary to other studies on recurrence, only patients with at least 1-year endoscopic follow-up were part of the analysis and in general patients are referred again when recurrence is encountered elsewhere, underestimation of recurrence numbers was prevented. Since data were collected in two experienced large tertiary centers one could state that the results may not be generalizable. However, we would like to emphasize that endoscopic papillectomy is an invasive low-volume procedure that should preferably be performed by experienced endoscopists only. Therefore, this cohort can be considered a good representation of the patient population and standard of practice. Finally, due to the long study period and involvement of two different centers the data could be subject to procedural changes overtime such as the use of NSAID and submucosal injection, snare types and the use of FCSEMS. Nonetheless, the principles of endoscopic papillectomy did not change significantly. Also, the fact that only patients who underwent endoscopic resection were included, excluding patients that were immediately referred for surgical review, should be considered when assessing this data. Moreover, evolution in imaging and understanding of the endoscopic features of malignancy may mean that contemporary rates of the endoscopic detection of malignancy are superior to the experience in this cohort.

Prospective long-term data need to confirm long-term risk on recurrence and (further) prove the benefit of, for example, the use of PD- and CBD-stents. However, this will be difficult due to low patient numbers. Furthermore, given the high percentage of unexpected malignancies, future studies should focus on methods to improve the endoscopic assessment of papillary lesions to prevent EP followed by PPPD in patients with a malignant lesion. Although, similar to Barrett’s neoplasia, endoscopic papillectomy may also be viewed as the definitive staging procedure, although the procedural risks are much greater and we would advise against this policy [30].

In conclusion, this long-term follow-up study shows a high level of recurrence, occurring even 5 years after successful endoscopic resection of a papillary adenoma. However, no adenomas underwent malignant degeneration during follow-up and endoscopic management can be considered adequate since surgery was prevented in the vast majority of cases. Due to low patient numbers it seems to be hard or impossible to conduct large prospective or randomized controlled trials with long-term follow-up. Therefore, retrospective data should be currently considered the best available evidence in this specific group of patients. As a result, we advise to consider at least 5-year follow-up in patients with a sporadic adenoma whose comorbidity or age does not make follow-up findings irrelevant. Longer follow-up should be strongly considered in young and fit patients and in case of FAP.

## Electronic supplementary material

Below is the link to the electronic supplementary material.

Supplementary file1 (DOCX 13 kb)

## Abbreviations

CBD: Common bile duct
CI: Confidence interval
CT: Computed tomography
EP: Endoscopic papillectomy
FAP: Familial adenomatous polyposis
FCSEMS: Fully covered self-expandable metal stent
HGD: High-grade dysplasia
IQR: Interquartile range
LGD: Low-grade dysplasia
NSAID: Non-steroidal anti-inflammatory drug
OR: Odds ratio
PD: Pancreatic duct
PPPD: Pylorus preserving pancreaticoduodenectomy
SD: Standard deviation
TA: Tubular adenoma
TVA: Tubulovillous adenoma
95% CI: 95% Confidence interval
