# Supplementary material for: Endoscopic papillectomy; a retrospective international multicenter cohort study with long-term follow-up
Source: Surg Endosc. 2020 Nov 6;35(11):6259–67. doi: 10.1007/s00464-020-08126-x (PMC8523407; doi:10.1007/s00464-020-08126-x)
Supplement: Supplementary file 1 — Supplementary file1 (DOCX 13 kb) [file 464_2020_8126_MOESM1_ESM.docx]

**Supplemental Table 1. Logistic regression analysis for factors associated with adverse events (n=259).**

|  | **Post-procedural bleeding** | **Pancreatitis** | **Papillary stenosis** |
| --- | --- | --- | --- |
| Variable | Univariate analysis  OR (95%CI) | Univariate analysis  OR (95%CI) | Univariate analysis  OR (95%CI) |
| Lesion size^a^ | 1.03 (0.999 – 1.053) | 0.98 (0.934 – 1.034) | 1.02 (0.988 – 1.052) |
| Piecemeal | 1.08 (0.492 – 2.363) | 1.56 (0.599 – 4.082) | 2.21 (0.858 – 5.703) |
| Malignancy | 0.665 (0.191 – 2.320) | - | 0.32 (0.041 – 2.432) |
| PD-stent | - | 1.31 (0.365 – 4.669) | 0.53 (0.191 – 1.461) |
| Intraductal extension | - | - | 1.61 (0.506 – 5.159) |

CI, confidence interval; OR, odds ratio; PD, pancreatic duct.
^a^Missing in 115 patients.
